# Effect of epileptiform discharges and hippocampal volume on cognitive dysfunction following clipping of ruptured aneurysms in the anterior circulation

**DOI:** 10.3389/fsurg.2025.1428311

**Published:** 2025-01-20

**Authors:** Kunitoshi Otsuka, Shigeki Sunaga, Hiroyuki Jimbo, Yoshinori Suzuki, Michihiro Kohno

**Affiliations:** ^1^ Department of Neurosurgery, Tokyo Medical University Hachioji Medical Center, Tokyo, Japan; ^2^ Department of Clinical Radiology, Tokyo Medical University Hachioji Medical Center, Tokyo, Japan; ^3^ Department of Neurosurgery, Tokyo Medical University Hospital, Tokyo, Japan

**Keywords:** aneurysmal clipping, cognitive dysfunction, interictal epileptiform discharges, hippocampal volume, spike index, subarachnoid hemorrhage

## Abstract

**Introduction:**

Cognitive dysfunction after aneurysmal subarachnoid hemorrhage (aSAH) remains unclear due to various neurological impairments. This study aimed to evaluate the changes in hippocampal volume, cognitive function, and interictal epileptiform discharges after clipping in patients with aSAH of anterior circulation.

**Methods:**

Patients with modified Rankin Scale scores of 0–3 points who underwent clipping were evaluated. Aneurysmal locations were classified as middle cerebral artery (MCA), internal carotid artery (ICA), and anterior cerebral artery (ACA). Surgery was performed using the transsylvian approach or interhemispheric approach. Hippocampal volume measurement, neuropsychological assessments, and interictal electroencephalogram evaluations were performed postoperatively at the subacute phase. Epileptiform discharges were assessed using the spike index (SI).

**Results:**

We included 60 patients (23 men, 37 women; median age, 57.4 years). Aneurysmal locations were found in the MCA, ICA, and ACA in 23, 19, and 18 patients, respectively. The postoperative hippocampal volume was significantly reduced on the clipping approach side in the MCA and ICA groups (MCA, *p* < .001; ICA, *p* < .001). There was no correlation between hippocampal volume and cognitive function. A significant difference was noted in elevated SI on the approach side of the MCA (*p* < .001), ICA (*p* < .001), and ACA (*p* < .001) in the transsylvian approach group. The elevated SI on the left approach side showed significant differences in some neuropsychological assessments (performance intellectual quotient, *p* = .028; perceptual organization, *p* = .045; working memory, *p* = .003).

**Discussion:**

Cognitive dysfunction in the subacute phase after clipping for aSAH was not correlated with hippocampal volume reduction but was correlated with interictal epileptiform discharges.

## 1 Introduction

Psychosocial problems after aneurysmal subarachnoid hemorrhage (aSAH) are common, even in patients classified as rehabilitated and those with a favorable prognosis, thus affecting the patient's quality of life (1). Temporomesial atrophic changes might be associated with cognitive impairment after aSAH (2, 3). In addition, Wostrack et al. (4) reported that hippocampal atrophy was substantially greater after clipping than coiling. Aneurysm surgery may trigger neurocognitive outcomes that are less favorable than those triggered by endovascular approaches (5, 6).

The postoperative seizure rate for aSAH varies from 2% to 11%, and subarachnoid hematoma and craniotomy may contribute to its development (7–9). Therefore, the early evaluation of cognitive function and interictal epileptiform discharges (IEDs) in electroencephalography (EEG) after aSAH is important to patient outcomes. However, the relationships between clipping, hippocampal damage, cognitive dysfunction, and epilepsy remain unclear.

This study aimed to evaluate the surgical effect on hippocampal volume, cognitive function, and IEDs in patients in the subacute phase who underwent clipping for aSAH of anterior circulation.

## 2 Materials and methods

Patients with subarachnoid hemorrhage (SAH) who experienced their first anterior circulation aneurysm rupture between 2010 and 2018 and with modified Rankin Scale scores of 0–3 points at 1 month after undergoing clipping at our hospital were included. This study was approved by the Ethics Committee at Showa University (Approval No. 2972) and was conducted in accordance with the ethical standards of the 1964 Declaration of Helsinki and its later amendments. Informed consent was obtained from all participants. We classified the locations of the ruptured aneurysm into the middle cerebral artery (MCA), internal carotid artery (ICA), and anterior cerebral artery (ACA) areas. Aneurysms in the MCA and ICA areas were clipped via the transsylvian approach (TSA) ipsilateral to the ruptured aneurysm, and those in the ACA area were clipped via the TSA or interhemispheric approach (IHA). Right craniotomy was performed for all patients undergoing the IHA. All patients underwent surgery immediately after diagnosis. Fluid management was implemented in all patients to maintain normovolemia 3 days to 2 weeks after craniotomy and clipping, in addition to fasudil hydrochloride administration via drip infusion. Cilostazol and statins were administered orally to prevent cerebral vasospasm. A ventriculoperitoneal shunt was placed in patients who developed hydrocephalus.

Patients with modified Rankin Scale scores of 0–3 points underwent magnetic resonance imaging (MRI) of the head, neuropsychological assessments, and interictal EEG evaluations postoperatively. The three-dimensional hippocampal volume was measured using MRI, and the volumes of the bilateral hippocampi were compared. The relationships of the hippocampal volume and neuropsychological assessments with the presence of intracerebral hematoma, intraventricular hematoma, delayed cerebral vasospasm, and hydrocephalus were also evaluated in the imaging studies. Furthermore, postoperative MRI fluid-attenuated inversion recovery (FLAIR) with high intensity on the brain cortex of the approach side was defined as FLAIR high-signal intensity. Thus, the FLAIR high-signal intensity would indicate the effects of SAH, circulatory disturbances in the brain, and cerebral damage by surgical manipulation. The relationships between the neuropsychological assessment and EEG findings and the presence of FLAIR high-signal intensity were evaluated.

### 2.1 Hippocampal volume measurement

The hippocampal volume was measured postoperatively using a 1.5-T MRI system (MPRAGE, FOV: 220/186/179; voxel size: 0.43 × 0.43 × 0.86 mm; TR/TE: 1220/4.29; repetition time: 1,160 ms; echo time: 4.27 ms; matrix: 256′256 pixels; MAGNETOM Advance, Siemens Healthcare, Erlangen, Germany) according to the method described by Pruessner et al. (10) Three-dimensional T1-weighted imaging (magnetization prepared rapid acquisition with gradient echo) was performed along the transverse, coronal, and sagittal planes. The hippocampal margin was traced manually in each plane, and the hippocampal volume was determined by reconstituting the data in three dimensions using an image analysis workstation (Ziostation2, Ziosoft, Inc., Tokyo). Measurements were performed by a neurosurgeon and radiologist, and the hippocampal volume was obtained.

### 2.2 Cognitive functions

Neuropsychological assessments were performed in accordance with the intelligence quotient measured using the Japanese version of the Wechsler Adult Intelligence Scale-Third Edition (WAIS-III) (11). Memory testing was measured using the Japanese version of the Wechsler Memory Scale-Revised (WMS-R) (12). The WAIS-III assessed Verbal Intellectual Quotient, Performance Intellectual Quotient, Full-scale Intellectual Quotient, Verbal Comprehension, Perceptual Organization, Working Memory, and Processing Speed, and the WMS-R assessed verbal memory, visual memory, general memory, Attention/Concentration and Delayed Recall. The correlations among these scores and hippocampal atrophy findings were examined.

### 2.3 IEDs in EEG

EEG assessments were performed interictally using a digital electroencephalograph (Nihon Kohden, Tokyo). The appearance of IEDs was examined at each aneurysm location using the spike index (SI) measurement method of Kessler et al. (13). The SI was expressed as the number of 1 s bins containing one or more epileptiform discharges (spike waves, sharp waves, and spike-and-slow wave complex), divided by the total number of seconds in the sleep recording and multiplied by 100 to calculate the percentage of the trace. The evaluation of SI was blinded for the aneurysmal location, the hippocampal volume, and the neuropsychological assessments. We measured SI on the approach and non-approach sides.

### 2.4 Statistical analysis

Statistical analysis was performed using SPSS version 27 (IBM Corp., Armonk, NY). Wilcoxon's signed rank-sum test was performed to assess the bilateral hippocampal volumes and SI values on the approach and non-approach sides. The Mann−Whitney *U*-test was used to examine the relationships of radiological findings, such as intracerebral hemorrhage, intraventricular hematoma, delayed cerebral vasospasm, hydrocephalus, and FLAIR high-signal intensity with the hippocampal volume on the approach side, neuropsychological assessments, and SI. The SI and cognitive function findings were compared among aneurysm locations using univariate analysis. The correlation between hippocampal volume and cognitive function or SI was examined using Pearson's product-moment correlation coefficient in the presence of a normal distribution and Spearman's rank correlation coefficient in the absence of a normal distribution. A correlation coefficient of ≥|0.5| denoted a correlation, whereas a correlation coefficient of ≥|0.7| denoted a strong correlation. The threshold for statistical significance was set at *p* < 0.05.

## 3 Results

**Figure 1:**
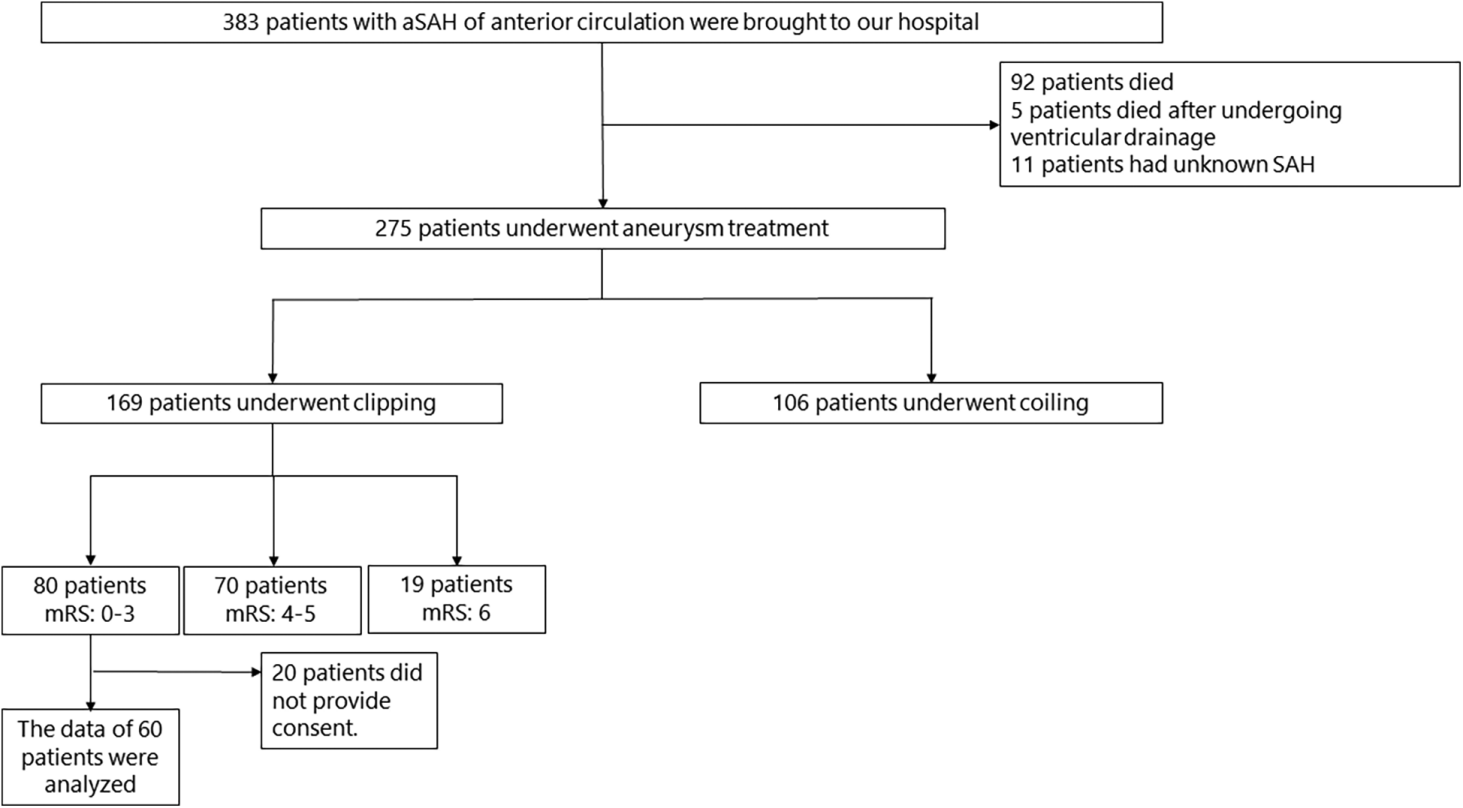
Flowchart of patients with aSAH. aSAH, aneurysmal subarachnoid hemorrhage; SAH, subarachnoid hemorrhage; mRS, modified rankin scale.

The flowchart of patients with aSAH and their clinical characteristics are presented in Figure 1 and Table 1, respectively. MRI, neuropsychological assessments, and EEG were performed in a median of 30 (27–46), 41 (27–80), and 32 (28–46) days, respectively.

**Table 1:** Clinical characteristics.

Characteristics		Data
Age (years)		57.4 (range 34.1–71.4)
Sex, female		37 (61.7%)
WFNS grade	Ⅰ	8 (13.3%)
	Ⅱ	26 (43.3%)
	Ⅲ	11 (18.3%)
	Ⅳ	12 (20.0%)
	Ⅴ	3 (5.0%)
Radiological findings at admission		
Fisher group	1	4 (6.7%)
	2	21 (35.0%)
	3	21 (35.0%)
	4	14 (23.3%)
Intracerebral hematoma		14 (23.3%)
Intraventricular hemorrhage		10 (16.7%)
Aneurysm location		
MCA area (*n* = 23)		
TSA	Right	11
	Left	12
ICA area (*n* = 19)		
TSA	Right	15
	Left	4
ACA area (*n* = 18)		
TSA	Right	8
	Left	2
IHA		8
Radiological findings		
Delayed cerebral vasospasm		15 (25.0%)
Hydrocephalus		9 (15.0%)
FLAIR high-signal-intensity		
MCA		12 (52.2%)
ICA		12 (63.2%)
ACA		12 (66.7%)
Outcome mRS	0	9 (15.0%)
	1	23 (38.3%)
	2	18 (30.0%)
	3	10 (16.7%)
Focal impaired awareness seizure (*n* = 12)		
MCA area		5
ICA area		4
ACA area		3

WFNS, World Federation of Neurological Surgeons; MCA, middle cerebral artery; ICA, internal carotid artery; ACA, anterior cerebral artery; TSA, transsylvian approach; IHA, interhemispheric approach; FLAIR, fluid-attenuated inversion recovery; mRS, modified rankin scale.

### 3.1 Evaluation of the hippocampal volume

No correlation was found between the number of clips and hippocampal volume at all aneurysm locations. Clipping was performed via the TSA ipsilateral to the ruptured aneurysm in all patients in the MCA and ICA groups. In patients with right aneurysm, the mean right and left hippocampal volumes were 3.11 ± 0.79 cm^3^ and 3.61 ± 0.52 cm^3^ and 3.19 ± 0.53 cm^3^ and 3.53 ± 0.46 cm^3^ in the MCA and ICA groups, respectively. In patients with left aneurysm, the mean right and left hippocampal volumes were 3.74 ± 0.77 cm^3^ and 3.30 ± 0.56 cm^3^ and 3.70 ± 0.29 cm^3^ and 2.80 ± 0.96 cm^3^ in the MCA and ICA groups, respectively. In patients in the ACA group who underwent surgery via the right TSA, the right and left hippocampal volumes were 2.89 ± 0.91 cm^3^ and 2.94 ± 0.89 cm^3^, respectively. In those who underwent surgery via the left TSA, the right and left hippocampal volumes were 2.55 ± 0.02 cm^3^ and 2.31 ± 0.11 cm^3^, respectively. In patients who underwent surgery via the IHA, the right and left hippocampal volumes were 3.48 ± 0.49 cm^3^ and 3.33 ± 0.60 cm^3^, respectively.

**Figure 2:**
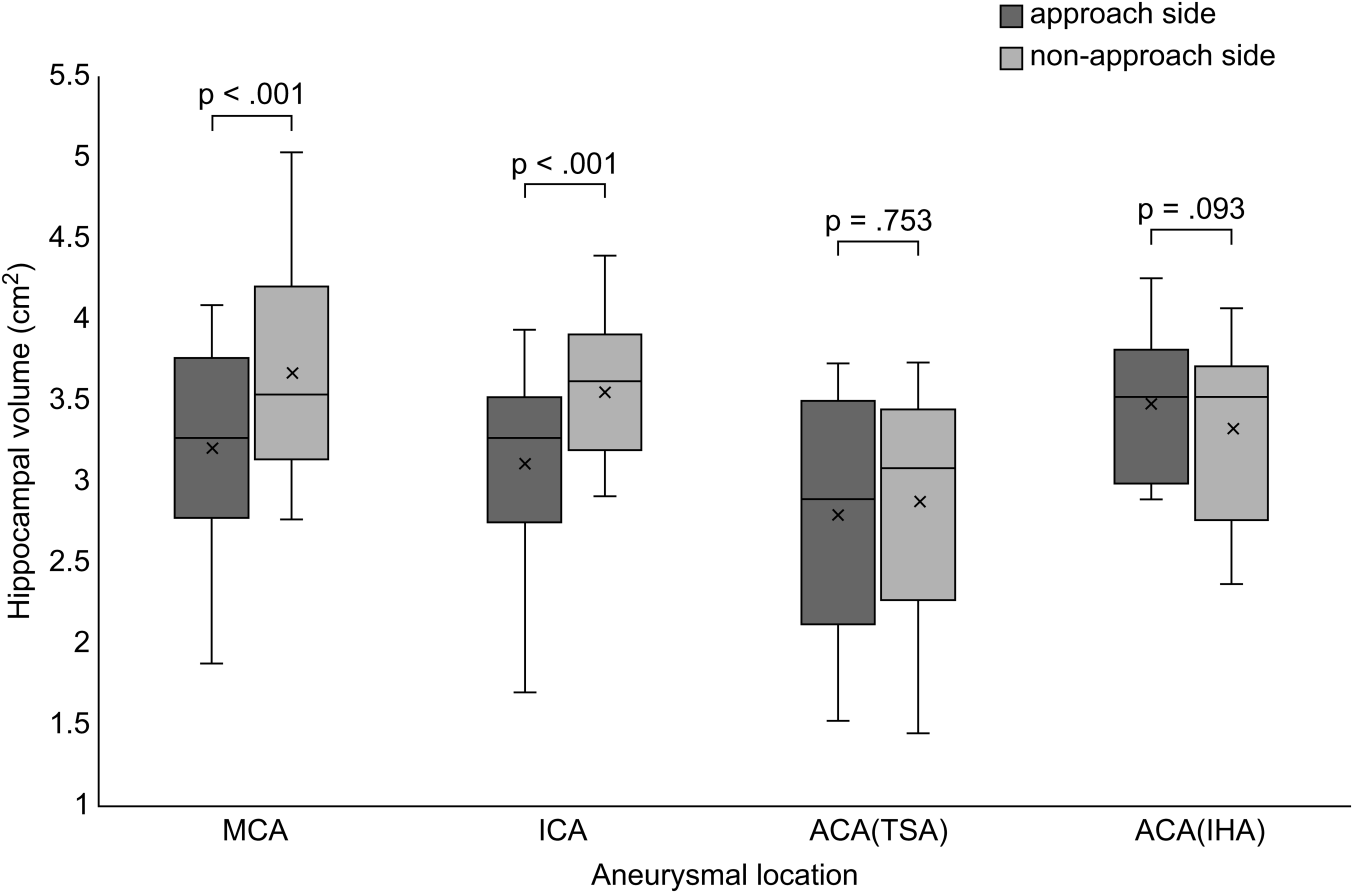
Comparison of hippocampal volume between the approach and non-approach sides after clipping surgery. Box and whisker plots show the distribution of hippocampal volume for each aneurysmal location. MCA, middle cerebral artery; ICA, internal cerebral artery; ACA, anterior cerebral artery; TSA, transsylvian approach; IHA, interhemispheric approach.

The hippocampal volume ipsilateral to the approach side was smaller than that ipsilateral to the non-approach side postoperatively in the MCA and ICA groups (MCA, *p* < .001; ICA, *p* < .001). No significant difference was observed between the left and right hippocampal volumes postoperatively in the ACA group (TSA, *p* = .753; IHA, *p* = .093) (Figure 2). The presence of radiological findings, such as intracerebral hemorrhage, intraventricular hematoma, delayed cerebral vasospasm, and hydrocephalus, was not related to the hippocampal volume on the approach side in the MCA, ICA, and ACA groups.

### 3.2 Evaluation of cognitive function

There was no significant difference in the cognitive function among aneurysm locations. No correlations were observed between cognitive function and hippocampal volume in the MCA, ICA and ACA areas. The hippocampal volume in the left and right approach sides was not correlated with cognitive function at any aneurysmal location. ACA showed significant differences between FLAIR high-signal intensity in working memory (*p* = .04) and general memory (*p* = .036), but no other significant differences were found (Table 2).

**Table 2:** Relationship between the radiological findings of aneurysmal location and neuropsychological assessment.

		WAIS-III							WMS-R				
		VIQ	PIQ	FIQ	VC	PO	WM	PS	Verbal memory	Visual memory	General memory	Attention/concentration	Delayed recall
MCA area	ICH	.46	.538	.854	.624	.327	.462	.854	.925	.573	.743	.083	.743
	IVH	.276	.384	.347	.148	.563	.885	.426	.71	.136	.832	.632	.49
	DCV	.805	.389	.462	.806	.426	.54	.65	.511	.888	.639	.542	.815
	Hydrocephalus	.798	.609	.552	.862	.552	.308	.67	.085	1.00	.138	.259	.123
	FLAIR high-signal-intensity	.236	.214	.215	.157	.288	.814	.059	.331	.965	.48	.565	.507
ICA area	ICH	—*	—*	—*	—*	—*	—*	—*	—*	—*	—*	—*	—*
	IVH	—*	—*	—*	—*	—*	—*	—*	—*	—*	—*	—*	—*
	DCV	.847	.578	.784	.848	.941	.804	.253	.628	.582	.669	.329	.409
	Hydrocephalus	—*	—*	—*	—*	—*	—*	—*	—*	—*	—*	—*	—*
	FLAIR high-signal-intensity	.361	.308	.415	.071	.359	.251	.181	.697	.116	.31	.508	.434
ACA area	ICH	.298	.296	.298	.292	.276	.71	.251	.216	.298	.328	.404	.15
	IVH	.494	.288	.298	.298	.285	.733	.225	.361	.79	.678	.92	.427
	DCV	.557	.77	.883	1.0	.769	1.0	.378	.432	.513	.432	.694	.359
	Hydrocephalus	.222	.222	.222	.222	.222	.222	.444	.2	.2	.2	.2	.2
	FLAIR high-signal-intensity	.078	.143	.142	.142	.142	.04	.557	.089	.067	.036	.513	0.116

MCA, middle cerebral artery; ICA, internal carotid artery; ACA, anterior cerebral artery; ICH, intracerebral hemorrhage; IVH, intraventricular hemorrhage; DCV, delayed cerebral vasospasm; FLAIR, fluid-attenuated inversion recovery; WAIS-III, Wechsler adult intelligence scale-third edition; WMS-R, Wechsler memory scale-revised; VIQ, verbal intellectual quotient; PIQ, performance intellectual quotient; FIQ, full-scale intellectual quotient; VC, verbal comprehension; PO, Perceptual Organization; WM, working memory; PS, processing speed.

The table shows the *p*-values obtained using the Mann−Whitney *U*-test.

* The *p*-value could not be determined in the ICA area because there were no patients with either ICH, IVH, or hydrocephalus in this group.

### 3.3 Evaluation of spike index and postoperative seizure

**Figure 3:**
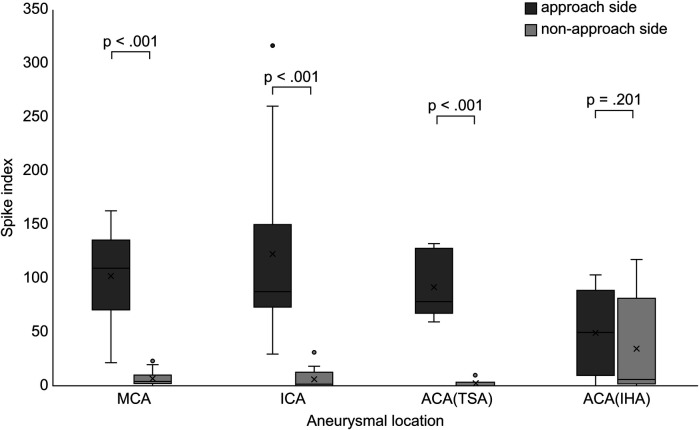
Comparison of the spike index between the approach and non-approach sides after clipping surgery. Box and whisker plots showing the distribution of the SI for each aneurysmal location. MCA, middle cerebral artery; ICA, internal cerebral artery; ACA, anterior cerebral artery; TSA, transsylvian approach; IHA, interhemispheric approach; SI, spike index.

There was no significant difference in the SI of the approach side among aneurysm locations (*p* = .34). SI was significantly elevated on the approach side in the MCA group (*p* < .001), ICA group (*p* < .001), and ACA subgroup treated via the TSA (*p* < .001) (Figure 3). No significant correlation was found between the hippocampal volume and SI on the approach side (MCA, *p* = .997; ICA, *p* = .209; ACA, *p* = .207) (Table 3). The correlations between elevated SI values and cognitive function were found in working memory in the MCA (correlation coefficient: −0.642, *p* = .018) and visual memory in the ACA (correlation coefficient: −0.605, *p* = .084). Among all aneurysmal locations, no correlation was found between elevated SI values on the right approach side and cognitive function. However, elevated SI values on the left approach side showed some effects on performance intellectual quotient, perceptual organization, and working memory (correlation coefficients, 0.76, 0.718, and 0.891, respectively; *p* = .028, .045, .003, respectively) (Table 4). SI was not related to the presence of FLAIR high-signal intensity (Table 5). The FLAIR high-signal intensity was examined separately for frontal and temporal lobes in relation to SI, but no correlation was found. At 1 month postoperatively, there were 12 cases with focal impaired awareness seizure as the primary attack.

**Table 3:** Correlation between hippocampal volume and spike index on the approach side of the aneurysmal location.

Approach side of the aneurysmal location	Hippocampal volume (cm^3^)^a^	SI^a^	Coefficient	*p*-value
MCA	3.204 ± 0.666	102.1 ± 40.5	−0.001	0.997
ICA	3.108 ± 0.622	122.8 ± 79.7	0.552	0.174
ACA	3.135 ± 0.75	74.0 ± 40.0	−0.5	0.207

MCA, middle cerebral artery; ICA, internal carotid artery; ACA, anterior cerebral artery; SI, spike index.

^a^ Mean ± standard deviation.

**Table 4:** Correlation between the spike index and neuropsychological assessments of all aneurysmal locations.

		WAIS-III								WMS-R				
		VIQ	PIQ	FIQ	VC	PO	WM	PS	Verbal Memory		Visual Memory	General Memory	Attention/Concentration	Delayed Recall
SI on the Rt app.	Coefficient	−0.157	−0.246	−0.236	−0.103	−0.144	−0.259	−0.093	−0.036		−0.055	−0.042	−0.045	−0.016
	*p*-value	.485	.269	.291	.649	.522	.244	.681	.866		.799	.847	.833	.939
SI on the Lt app.	Coefficient	−0.608	−0.762	−0.703	−0.524	−0.718	−0.891	−0.707	−0.232		−0.256	−0.368	−0.334	−0.457
	*p*-value	.109	.028	.052	.183	.045	.003	.05	.447		.399	.216	.265	.116

WAIS-III, Wechsler adult intelligence scale-third edition; WMS-R, Wechsler memory scale-revised; VIQ, verbal intellectual quotient; PIQ, performance intellectual quotient; FIQ, full-scale Intellectual Quotient; VC, verbal comprehension; PO, Perceptual Organization; WM, working memory; PS, processing speed; app., approah; SI, spike index.

**Table 5:** Relationship between the spike index (SI) and the presence of FLAIR high-signal intensity.

	SI^a^	FLAIR high-signal intensity	*p*-value
MCA area	102.1 ± 40.5	52.2%	.288
ICA area	122.8 ± 79.7	63.2%	.23
ACA area	74.0 ± 40.0	66.7%	.451

MCA, middle cerebral artery; ICA, internal carotid artery; ACA, anterior cerebral artery; FLAIR, fluid-attenuated inversion recovery; SI, spike index.

^a^ Mean ± standard deviation.

## 4 Discussion

We investigated the changes in brain structure, electrical activity, and cognitive function after clipping for aSAH of anterior circulation. In terms of brain structural changes, we focused specifically on the hippocampus and found that the hippocampal volume was reduced on the approach side compared to the non-approach side. Furthermore, regarding the relationship between hippocampal volume reduction and cognitive function, no correlations were observed in the MCA, ICA and ACA areas. SI was significantly elevated on the approach side in TSA, and elevated SI on the left approach was strongly correlated with a decline in cognitive function. There was no correlation between elevated SI and brain structural changes, such as hippocampal volume reduction and FLAIR high-signal intensity.

Hippocampal atrophy after clipping has been reported previously. Inoue et al. (14) reported that after surgery for an unruptured cerebral aneurysm, an enlarged inferior horn was observed on MRI due to intraoperative microvascular insufficiency and cerebral compression. Wostrack et al. (4) showed that patients had greater hippocampal atrophy after clipping than after endovascular treatment. Meanwhile, the relationship between intracerebral hemorrhage and hippocampal atrophy has been previously reported (15). The mechanism is that the medial structures of the temporal lobe are in contact with the tentorial incisura, making the hippocampus vulnerable to the effects of increased intracranial pressure (16). Therefore, local intracranial hypertension, insufficiency of the intracranial circulation, and the anatomical structure of the hippocampus and subarachnoid hemorrhage may lead to apoptosis of hippocampal neurons, thereby causing hippocampal volume reduction after aSAH. Surgery for aSAH is more difficult than surgery for unruptured aneurysms due to cerebral edema, which makes it more difficult to secure an adequate surgical field. Thus, microvascular insufficiency due to retraction of the temporal lobe may cause hippocampal volume reduction, and surgical manipulation/approach may be a factor that causes hippocampal volume reduction. The reduction in hippocampal volume on the operative side may also be caused by the force of the hemorrhage itself. The lack of left-right differences in hippocampal volume in ACA aneurysms may be related to the fact that the force of hemorrhage is not distinctly lateralized, as the force of hemorrhage often spreads anteriorly or posteriorly.

Regarding the timing of hippocampal atrophy, Wostrack et al. (4) assessed the extent of atrophy at 17 months postoperatively; herein, it was observed at 1 month postoperatively. This suggests that postoperative hippocampal volume reduction may occur earlier than previously thought.

The cause of cognitive dysfunction after aSAH remains unclear because of the diversity of neurological disorders. In general, mild to moderate cognitive dysfunction may reflect secondary brain damage due to intracranial circulatory disorder and subarachnoid hematoma (17, 18). Inoue et al. (14) reported no evidence of cognitive dysfunction in patients who presented with dilation of the inferior horns after clipping. However, sub-analyses of the International Subarachnoid Aneurysm Trial reported that cognitive dysfunction and epileptogenesis occur significantly less often after endovascular surgery (19, 20). These suggest that patients are more liable to epileptic seizures and cognitive dysfunction after clipping.

There was no correlation between elevated SI and brain structural changes on MRI in our study. Lüders et al. (21) reported on a network that develops epilepsy, suggesting that the elevated SI in the 1-month postoperative period of our study may complement interictal epileptiform discharges and capture the irritative zone they advocated. Jensen et al. (22) mentioned that morphological changes in the network reorganization that acquire epileptogenesis occur within weeks to months after injury. It is possible that the epileptogenesis was not acquired because the timing of our SI assessment was a subacute phase.

Lv et al. (23) reported that patients with an IEDs index of >10% showed a decline in cognitive function. Similarly in our study, cognitive dysfunction was associated with IEDs in EEG rather than with hippocampal volume reduction. Binnie et al. (24) reported that epileptiform discharges are not confined to epilepsy and that cognitive function improves when epileptiform discharges are suppressed with anti-seizure medications. Elevated SI is an early indicator of possible future epileptic seizures, and a decision to use anti-seizure medications to control elevated SI may prevent the worsening of cognitive function.

### 4.1 Limitations

The main limitation of this study is the small sample size. The control group for hippocampal volume and SI was on the non-approach side, and future comparisons with the endovascular treatment group are desired. The study was limited to the subacute phase, and further investigation over time is needed to determine the structure and electrical activity of the cerebrum, cognitive function, and the occurrence of epileptic seizures in this population.

### 4.2 Conclusion

Hippocampal volume reduction and elevated SI were observed on the approach side in the subacute phase after clipping for the aSAH of anterior circulation. There was no association between cognitive dysfunction and hippocampal volume reduction. However, cognitive dysfunction was associated with IEDs.

## Data Availability

The original contributions presented in the study are included in the article/Supplementary Material, further inquiries can be directed to the corresponding author.
